# Immediate Effect of Four Exercises on Linea Alba Thickness, Distortion and Inter‐Recti Distance in Parous Women

**DOI:** 10.1002/pri.70185

**Published:** 2026-03-07

**Authors:** Montserrat Rejano‐Campo, Laura Fuentes‐Aparicio, Bernadette de Gasquet

**Affiliations:** ^1^ Departamento de Fisioterapia Facultad de Ciencias de la Salud Grupo de Investigación Prehabilitación y Rehabilitación de las disfunciones del movimiento, rendimiento, participación y calidad de vida Universidad Fernando Pessoa Canarias Las Palmas Spain; ^2^ Physiotherapy in Motion, Multi‐Speciality Research Group (PTinMotion) Department of Physiotherapy Faculty of Physiotherapy University of Valencia Valencia Spain; ^3^ De Gasquet Institute Paris France

**Keywords:** diastasis recti and weakness of the linea alba, exercise, physical therapy modalities, postpartum period

## Abstract

**Background and Purpose:**

This cross‐sectional study assessed the impact of four abdominal exercises (crunch, abdominal drawing‐in maneuver [ADIM], pelvic floor muscle contraction, and de Gasquet basic exercise) on inter‐recti distance (IRD) and linea alba (LA) distortion and thickness in parous women.

**Methods:**

Parous women underwent ultrasound assessment during four abdominal exercises. The primary outcome was IRD at supra‐ and infraumbilical levels. Secondary outcomes were LA distortion and thickness. Transversus abdominis (TrA) activation was assessed simultaneously. Exercises were compared using linear mixed‐effects models and Tukey‐adjusted pairwise tests.

**Results:**

Fifty‐four parous women participated. Supraumbilically, the crunch showed the greatest IRD decrease (EMM + 2.48 mm), but this was not significantly different from ADIM, pelvic floor muscle contraction, or de Gasquet exercise (Tukey *p* ≥ 0.07). The crunch was associated with increased LA distortion during the exercise compared with other exercises (supra: + 0.26 ± 0.05; infra: + 0.35 ± 0.07 distortion units; both *p* < 0.001), while the other three exercises reduced or maintained distortion during the exercise. Supraumbilical LA thickness increased during crunch compared with the other exercises (Tukey *p* ≤ 0.05). TrA effects were exercise‐specific: greater TrA thickening was associated with greater IRD decrease during ADIM, whereas lower TrA thickening was associated with greater IRD decrease during crunch.

**Discussion:**

The crunch showed the largest supraumbilical immediate reduction in IRD but was accompanied by increased LA distortion. In contrast, the other three exercises were associated with smaller IRD changes and lower distortion. Lower TrA activation during crunch was associated with increased LA deformation, and apparent medial convergence of the rectus abdominis during the exercise.

**Trial Registration:**

This research was registered on ClinicalTrials (NCT04501302)

## Implications for Physiotherapy Practice

- During the exercise, the crunch tended to show the greatest immediate reduction in supraumbilical inter‐recti distance; however, this change was not significantly different from abdominal drawing‐in, pelvic floor muscle contraction, or de Gasquet exercises, and was accompanied by increased linea alba distortion.
- Lower transversus abdominis thickening during crunch was associated with greater inter‐recti distance decrease and increased linea alba distortion, suggesting an association between transversus abdominis activation and linea alba mechanical behavior during this exercise.
- De Gasquet exercise showed mechanical responses similar to abdominal drawing‐in maneuver and pelvic floor muscle contraction, with limited distortion and preservation of linea alba thickness and inter‐recti distance.
- Observed variations in linea alba behavior and transversus abdominis activation during the exercises indicate exercise‐specific immediate mechanical responses and provide a basis for individualized assessment and future research.

**Abbreviations**
BMI: body mass index
IRD: interrectus distance
LA: Linea alba

## 1 Introduction

Diastasis rectus abdominis (DRA) is defined by the European Hernia Society as an abnormal separation of the two rectus abdominis muscles caused by a thinning and widening of the linea alba (LA) (Hernández‐Granados et al. 2021). DRA is associated with a negative body image perception (Keshwani et al. 2018), a decrease in quality of life (Gitta et al. 2017), the presence of gastrointestinal symptoms (Vicente‐Campos et al. 2022), and a decrease in abdominal muscle strength (Gunnarsson et al. 2015). This reduction in abdominal muscle force could be attributed to the lack of tension of the LA associated with its excessive elongation, potentially compromising optimal abdominal wall function (Lee and Hodges 2016), as the LA facilitates the transfer of forces within the trunk and pelvis (Mota et al. 2015). Transversus abdominis muscle (TrA) activation has been shown to have the capacity of tensioning the LA (Lee and Hodges 2016) and its training could be a key element for trunk stability in patients with DRA. Lee and Hodges developed the distortion index to measure tensioning in the LA (Lee and Hodges 2016). Nevertheless, this tension has rarely been explored in the literature, as most studies have used inter‐recti distance (IRD) as the primary and only outcome for the evaluation of DRA (Hills 2017; Lee and Hodges 2016). Other outcomes, such as LA thickness, could also be relevant in evaluating patients with DRA but are seldom used. Evaluating LA thickness during exercise is particularly interesting since excessive elongation of the LA could correlate with a decrease in tissue thickness and an increased risk of hernia.

Several cross‐sectional studies have assessed the behavior of the LA during the performance of abdominal exercises commonly used for treating of patients with DRA, most evaluating crunch and abdominal drawing‐in maneuvers (ADIM) (Boxer and Jones 1997; Chiarello and McAuley 2013; Gluppe et al. 2020; Keshwani et al. 2018; Lee and Hodges 2016; Mota et al. 2015; Pascoal et al. 2014; Sancho et al. 2015). However, most prior studies have focused on IRD alone; very few have examined IRD together with LA tension or distortion, and none have included LA thickness. Notably, Beamish et al. and Arranz‐Martín et al. extended beyond IRD to evaluate LA distortion, providing initial evidence on how different tasks influence the tensile behavior of the LA. Arranz‐Martín et al. compared distortion across a hypopressive abdominal exercise, an ADIM, a semi–curl‐up, and an ADIM plus semi–curl‐up. The hypopressive protocol involved performing a specific posture: axial elongation of the trunk with hips and knees flexed, and wrist and ankle dorsiflexed, combined with an expiratory apnea emphasizing lateral rib expansion. In France, it is common for clinicians to use the de Gasquet method, which similarly prioritizes axial elongation and breath control to facilitate TrA activation. This method focuses on postural alignment by lengthening the spine and includes a contraction of the pelvic floor muscles (PFM) accompanied by exhalation, which facilitates the TrA contraction (Gasquet 2009; Gasquet and Astrid 2018).

Given the limited research assessing IRD, LA tension/distortion, and LA thickness together, and their simultaneous association with TrA activation during commonly prescribed exercises for DRA, our primary objective is (1) to compare the immediate effect of four exercises (crunch, ADIM, PFM contraction, de Gasquet basic exercise) on IRD at supra‐ and infraumbilical levels. Our secondary objectives are (2) to compare LA distortion index across exercises, (3) to compare LA thickness across exercises, and (4) to explore associations between TrA thickening and IRD, LA distortion, and LA thickness, in a group of parous women.

We hypothesize that exercises including a TrA activation (such as ADIM or PFM contraction) will increase the IRD but also be able to properly tension the LA, without causing excessive thinning. Conversely, exercises that focus on rectus abdominis contraction (such as the abdominal crunch) will decrease the IRD but also increase LA distortion during the exercise. Combined exercises (such as the de Gasquet exercise) may tension the LA without increasing IRD or decreasing its thickness. We also expect that greater TrA thickening will be associated with less distortion and preserved thickness in all exercises.

## 2 Methods

### 2.1 Design

The present study is a monocentric cross‐sectional study performed between September 2020 and February 2021. The Ethical Committee *Comité de Protection des Personnes Ouest III* (20.01.16.71857) approved this study, which meets the criteria established by the Declaration of Helsinki. This research was registered on ClinicalTrials (NCT04501302) and was reported according to the STROBE guidelines (Vandenbroucke et al. 2007).

### 2.2 Participants

Parous women were recruited at the *Institute de Gasquet* in Paris, through the distribution of flyers within the Institute. The inclusion criteria were French‐speaking parous adult females who registered in the French Social Security system. Women were excluded if they had cognitive limitations in understanding questionnaires or exercises, or had a history of spinal, abdominal, and urogynecology surgery, any neuromuscular disease or sensitivity disorders. All participants signed an informed consent form prior to participation in the study.

### 2.3 Procedure

Participants attended one session at the *Institute de Gasquet*. Two evaluators (MRC and BG) with more than 10 years of experience in ultrasound imaging and DRA management, performed the evaluations. Initially, MRC explained the study to the women and collected sociodemographic and clinical data: age, BMI, number and type of deliveries, type of exercise and frequency. Subsequently, the women were asked to complete the Urinary Symptom Profile (USP) questionnaire in order to evaluate potential urinary incontinence symptoms (Haab et al. 2008). Participants were also asked to indicate potential low back pain severity at three periods: at the time of the examination, an average during the last week, and an average over the last 6 months, using a Visual Analog Scale (VAS, 0–100) (Delgado et al. 2018). Lastly, all women underwent two simultaneous ultrasound imaging assessments. Two ultrasound scanners (Mindray M7 and Essaote Mylab Gamma) were placed into two different locations and recorded independently:

— One linear probe was placed in the lower quadrant abdomen, midway between the costal margin and the iliac crest along the anterior axillary line, in order to capture abdominal TrA and internal oblique thickness (at rest and during activation).
— Simultaneously, another linear probe was placed transversely along the midline of the abdomen. Two locations of the LA were chosen based on commonly used anatomical reference points reported in the literature: 3 cm above the umbilicus and 2 cm below the umbilicus (Keshwani et al. 2018; Nahas et al. 2001).

To enhance consistency between the two ultrasound devices, probe placement, participant positioning, breathing phase, acquisition protocol, and image‐analysis procedures were standardized across devices.

**FIGURE 1:**
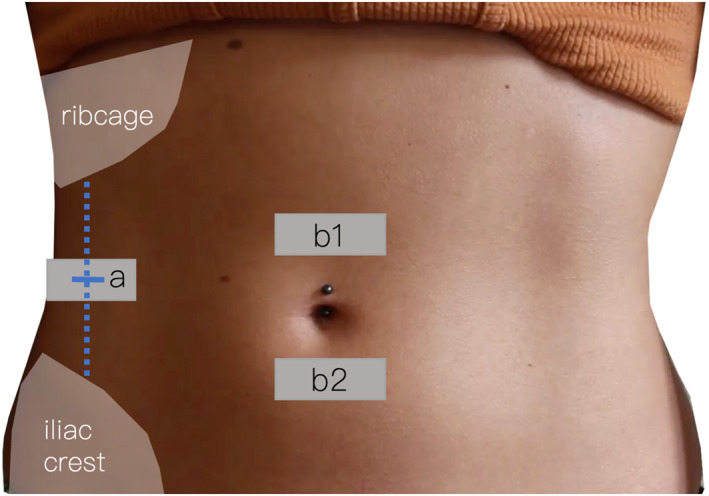
Probe placement and measurement sites. (a) Linear probe on the right lateral abdominal wall, midway between the costal margin and the iliac crest along the anterior axillary line, to record transversus abdominis and internal oblique thickness at rest and during activation. (b1) Supraumbilical linea alba site, 3 cm above the umbilicus. (b2) Infraumbilical linea alba site, 2 cm below the umbilicus. Sites a and b were acquired simultaneously.

Probe placement for the simultaneous ultrasound recordings is shown in Figure 1. MRC performed the ultrasound assessment of the LA, and BG performed the assessment of the lateral abdominal wall.

**FIGURE 2:**
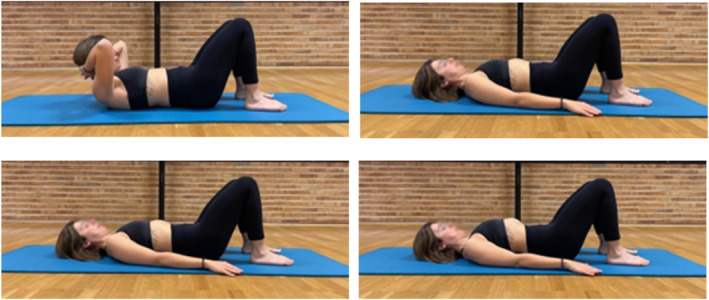
Illustration and description of the four exercises. Participants were instructed to exhale slowly and maintain the contraction at the end of expiration for approximately 3–5 s. Exhalation timing was visually monitored by the evaluator through observation of abdominal motion and verbally guided to ensure consistency across repetitions. Contraction intensity was standardized by instructing participants to perform a controlled submaximal voluntary contraction sufficient to execute the target exercise without compensatory trunk, pelvic, or rib cage movements. Superior left: Crunch. Participants lifted their head until the scapulae were off the table for 3–5 s. Superior right: Abdominal drawing‐in maneuver. Participants were asked to draw the navel toward the spine for 3–5 s. Inferior left: pelvic floor muscle contraction. Participants were asked to perform a pelvic floor muscle contraction, with the instruction of squeezing the anus and vagina during 3–5 s. Inferior right: De Gasquet basic exercise. Participant's pelvis was placed in counternutation, the head, by performing a slight traction, was also placed in self‐elongation by performing a slight traction. The participant was expected to automatically breath through an abdominal breathing pattern. He was then instructed to contract her pelvic floor before breathing out and then exhale, maintaining elongation of the spine and a small pressure of the occiput on the table.

For the assessment, participants lay in a supine position with knees and hips flexed and arms along the body. MRC demonstrated how to properly perform each of the four exercises (details provided in Figure 2): the crunch exercise, ADIM, a PFM contraction, and the basic exercise of the de Gasquet method. The order of the exercises was chosen randomly. Women performed each exercise six times: three times for the assessment at the supraumbilical level and three times at the infraumbilical level. A 1‐min rest was provided between exercises to allow adequate neuromuscular recovery and avoid fatigability, in line with previous evidence supporting rest periods equal to or up to twice the contraction time (Staniszewski et al. 2024).

### 2.4 Outcomes

We collected sociodemographic and clinical variables, including age, body mass index (BMI), education level, employment status, type of sport, parity, months since last delivery, mode of delivery, urinary symptoms, and presence of low back pain. For delivery‐related variables, parity was categorized as (nulliparous, primiparous, or multiparous) and months since the last delivery were recorded as a continuous variable. Urinary symptoms were assessed using the Urinary Symptom Profile (USP) score.

Continuous ultrasound videos were recorded for each exercise and site, and three repetitions were performed. Two evaluators analyzed the ultrasound images using ImageJ software (Schneider et al. 2012). For each repetition, the first evaluator selected a single frame representing the maximal change from rest position, defined as the frame showing the greatest variation in IRD, LA thickness, or distortion index during the contraction phase. Images were acquired at the end of relaxed expiration to ensure consistent respiratory conditions, as recommended for ultrasound assessment of abdominal wall muscles (Teyhen et al. 2007; Whittaker et al. 2007). Measurements were obtained from this frame for each repetition, and values were averaged across the three repetitions for analysis. The second evaluator independently reviewed all selected images and corresponding measurements to verify image quality, anatomical landmark identification, and measurement accuracy. When discrepancies or uncertainties were identified, both evaluators reviewed the image together and reached a consensus. All ultrasound videos were anonymized and coded (“exercise 1–4”), and presented in randomized order. The evaluator was blinded to the correspondence between coded videos and actual exercise type during the measurement process, and exercise identity was revealed only after completion of all measurements. The following measurements were taken at rest and during each exercise:

— IRD distance. The shortest distance between the two medial borders was measured (Chiarello and McAuley 2013; Mota et al. 2012).
— LA thickness. The thickness of the LA was measured at its narrowest point (Espinoza‐Bravo et al. 2025).
— Distortion index. The area between the LA trajectory and the shortest distance between the two medial borders of the rectus abdominis muscles was measured. The distortion index was calculated by dividing the value of this area by the shortest distance between the two medial borders of the rectus abdominis muscles (Espinoza‐Bravo et al. 2025)
— TrA thickness. The thickness of the TrA was measured 1 cm lateral to the *linea semilunaris.*
— Internal oblique thickness. The thickness of the internal oblique muscle was measured 1 cm lateral to the *linea semilunaris* (Manshadi et al. 2011; McMeeken et al. 2004).

### 2.5 Statistical Analysis

A priori power analysis was conducted using G*Power (v3.1.9.4), assuming a medium effect size (*f* = 0.25, *d* = 0.40), *α* = 0.05, and power = 0.95. The required sample size was 36; accounting for a 20% attrition rate, a minimum of 44 participants was determined.

Six linear mixed‐effects regression models were fitted, assuming Gaussian residuals. These models predicted the relationship between (1) supraumbilical IRD decrease during exercise, (2) infraumbilical IRD decrease during exercise, (3) supraumbilical distortion index during exercise, (4) infraumbilical distortion index during exercise, (5) supraumbilical LA thickness decrease during exercise, and 6) infraumbilical LA thickness decrease during exercise. Supra‐ and infraumbilical IRD were predefined as the primary outcomes. Linea alba distortion index and thickness were predefined as secondary outcomes to provide complementary information on connective tissue mechanical behavior during the exercises. Analyses examining associations between transversus abdominis thickening and linea alba outcomes were considered exploratory. To account for multiplicity in pairwise exercise comparisons, Tukey‐adjusted post hoc tests were applied within each model to control the family wise error rate.

Exercise condition was included as a fixed effect in all models. Participant ID was included as a random intercept to account for repeated measures within individuals, as each participant performed all exercises and measurements were obtained at both anatomical sites. Continuous covariates were age, BMI, parity (number of prior deliveries), and months since the last delivery, as well as VAS and USP scores. Categorical covariates were mode of delivery (vaginal without forceps/vaginal with forceps/cesarean), education level, employment status, and type of sport. To evaluate the potential influence of postpartum duration and baseline morphology, additional sensitivity analyses were performed, including stratified univariate comparisons across postpartum duration groups and inclusion of postpartum duration and baseline morphological measures as covariates in the mixed‐effects models.

To reduce the risk of overfitting and multicollinearity, variance inflation factor (VIF) was calculated, with values > 5 indicating high correlation. Initially, the association between each covariate and each dependent variable was assessed using univariate mixed‐effects regression models including participant ID as a random intercept. Only covariates showing an association with the outcome at *p* < 0.15 were considered for inclusion in multivariable models (Zuur and Ieno 2016). Model fitting began with error structure selection, followed by random structure adjustment (REML), and stepwise fixed effect refinement (ML). Final models were refitted with REML. Post hoc tests were conducted using the *emmeans* package (v1.7.5), and model comparison was based on corrected AIC. Assumptions were checked via residual plots and DHARMa diagnostics. An alpha level of 0.05 was used for all analyses, which were conducted with R software version 4.2.2. Most models (1, 2, 5, and 6) demonstrated a good fit of the residuals, meeting the necessary assumptions of linearity, normality, and the absence of multicollinearity and outliers. However, models 3 and four exhibited a poor fit of the residuals, failing to meet the assumptions of normality and homoscedasticity. Consequently, results from these models should be interpreted with caution. Because residual diagnostics indicated deviations from normality and heteroscedasticity for distortion index models, additional non‐parametric sensitivity analyses were conducted. Specifically, paired Wilcoxon signed‐rank tests with Bonferroni correction were performed to compare distortion index changes between exercises at supra‐ and infraumbilical levels. These analyses were used to assess the robustness of the mixed‐model findings.

## 3 Results/Findings

Fifty‐four parous women participated in our study and performed all four exercises. Sociodemographic data are presented in Table 1. Postpartum duration showed wide variability but was not associated with IRD decrease, LA thickness decrease, or distortion index measures. Sensitivity analyses including postpartum duration and baseline morphological measures as covariates did not alter the exercise‐related effects. Detailed results are provided in the supplementary material (Tables [Link], [Link], [Link]).

**TABLE 1:** Demographical and clinical outcomes.

	*N*	
Age, years, mean (SD)	54	42.35 (9.60)
BMI, kg/m, mean (SD)	54	21.57 (2.68)
Level of studies (%)	54	
Without studies		1.9
Secondary education		5.6
Bachelor		14.8
Master		61.1
PhD		16.7
Work situation (%)	54	
Employed		87
Unemployed		5.6
Student		1.9
Retired		3.7
Other		1.9
Type of sport (%)	54	
None		24.1
Low impact^a^		55.6
High impact^b^		20.4
Parity (%)	54	
1		25.9
2		46.3
3		22.2
4		5.6
Months since last delivery, mean (SD)	54	93.92 (99.86)
Mode of delivery (%)		
Vaginal without forceps		61.1
Vaginal with forceps		3.7
C‐section		35.2
Score USP, mean (SD)	54	4.00 (4.88)
Current pain, VAS, mean (SD)	54	4.15 (10.65)
Supraumbilical IRD at rest, mean (SD)	54	22.57 (8.32)
Supraumbilical thickness of the linea alba at rest, mm, mean (SD)	54	1.22 (0.58)
Supraumbilical area of deformation of the linea alba at rest, mm^2^, mean (SD)	54	0.29 (0.48)
Infraumbilical IRD at rest, mean (SD)	52	19.05 (10.40)
Infraumbilical thickness of the linea alba at rest, mm, mean (SD)	52	1.35 (0.83)
Infraumbilical area of deformation of the linea alba at rest, mm^2^, mean (SD)	52	0.24 (0.43)
TrA thickness at rest, mm, mean (SD)	51	2.74 (0.85)
IO thickness at rest, mm, mean (SD)	51	5.98 (3.20)

Abbreviations: BMI: Body mass index, IO: internal oblique muscle, IRD: inter‐recti distance, SD: Standard deviation, TrA: transversus abdominis muscle, USP: Urinary system profile, VAS: visual analog scale.

^a^ Walking, swimming, cycling, yoga, Pilates.

^b^ Running, musculation, fitness, high impact dance.

Intra‐rater reliability of the ultrasound measurements has previously been demonstrated to be good to excellent, with intraclass correlation coefficients (ICC) above 0.90 for IRD, LA thickness, and distortion index when repeated measurements were performed on the same selected images. Complete results and additional details are available in Espinoza‐Bravo et al. 2025(Espinoza‐Bravo et al. 2025). Comprehensive univariate analyses investigating the impact of independent variables on IRD, distortion index and LA thickness are reported in Table S3.

### 3.1 Type of Exercise

**FIGURE 3:**
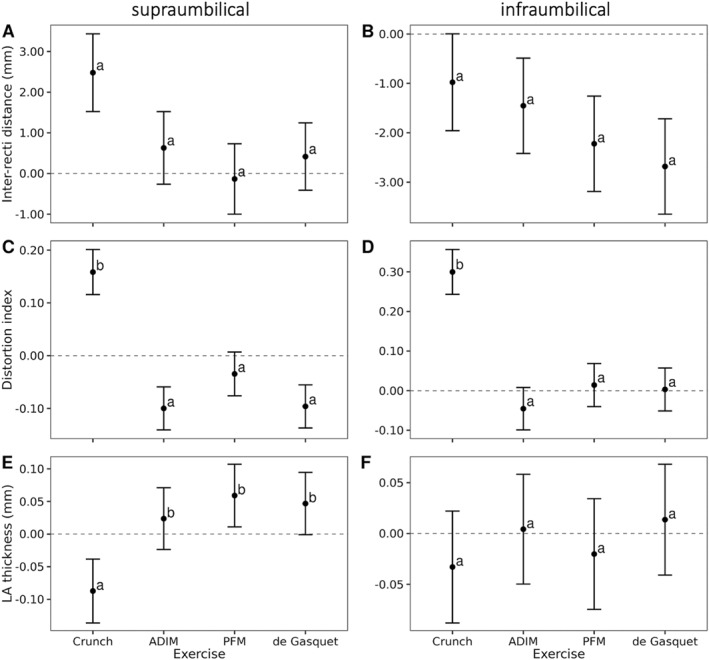
Comparison between exercises on inter‐recti distance, distortion index and linea alba thickness. LA: linea alba. Panels *A–C* correspond to the supraumbilical level and *D–F* to the infraumbilical level. *A–B* correspond to IRD decrease, *C–D* correspond to distortion index increase and *E–F* correspond to LA thickness decrease. Different letters (a, b) indicate statistically significant differences between exercises (*p* < 0.05). Error bars represent 95% confidence intervals. The crunch produced the greatest supraumbilical IRD reduction although this difference was not statistically significant across exercises (all labeled *a*). It also showed a significantly higher distortion index (*b*) and a significant increase in LA thickness (*a*) compared with the other exercises (*b*), which tended to maintain thickness. ADIM, PFM contraction, and the de Gasquet basic exercise exhibited similar IRD and distortion values, indicating minimal deformation of the linea alba. At the infraumbilical level, all exercises showed a nonsignificant tendency toward IRD reduction, with no significant differences in distortion or LA thickness. Values from these graphics can be consulted in Table S4.

The type of exercise influenced abdominal wall outcomes in the univariate analysis. It showed a significant association with IRD supraumbilically (Chi = 19.20, *p* < 0.001), LA thickness supraumbilically (Chi = 15.05, *p* = 0.002), and the distortion index at both levels (supraumbilical: Chi = 36.40, *p* < 0.001; infraumbilical: Chi = 43.18, *p* < 0.001). Figure 3 illustrates the multivariate analysis, while comprehensive details are available in the supplementary materials for reference (Table S4).

**FIGURE 4:**
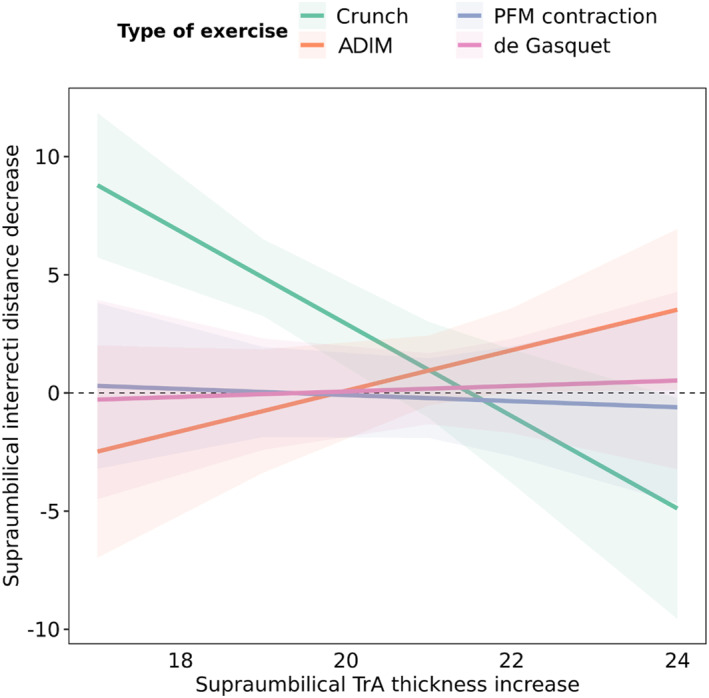
Interaction between supraumbilical inter‐recti distance decrease and supraumbilical thickness increase of the transversus abdominis muscle. Crunch (green): negative slope: when TrA thickening is lower, the IRD decreases. ADIM/Drawing‐in (red): positive slope: greater TrA thickening is associated with a larger IRD decrease. PFM contraction (purple) and de Gasquet (orange): near‐flat slopes with wide CIs around zero—no clear coupling between TrA thickening and IRD change. The shaded bands (CIs) indicate that the crunch and ADIM slopes differ in direction, supporting a significant exercise × TrA interaction; PFM and de Gasquet show nonsignificant trends. ADIM: Abdominal drawing‐in maneuver; TrA: Transversus abdominis muscle.

1. IRD: No significant differences in IRD change were found between exercises (Tukey *p* ≥ 0.07, Figure 3A,B). Specifically, supraumbilically, crunch showed the greatest IRD decrease during the exercise (2.48 ± 0.96 mm, 95% CI 0.59–4.37), whereas ADIM, PFM and de Gasquet exercise showed changes between −0.13 and 0.63 mm (details in Table S4). All four exercises exhibited negative IRD decrease values (Figure 4b), indicating widening relative to rest, with no significant between‐exercise differences (Tukey: all pairwise *p* = 0.34–0.97). Infraumbilically, all exercises showed negative IRD decrease values, indicating widening relative to rest. Mean changes ranged from −0.98 ± 0.98 mm during crunch to −2.68 ± 0.97 mm during the de Gasquet exercise, with no significant differences between exercises (Tukey: all pairwise *p* = 0.34–0.97).
2. Distortion: The reduction in IRD observed supraumbilically during the crunch was accompanied by an increase in distortion index during the exercise (+0.16 ± 0.04, 95% CI 0.07–0.24), whereas the other three showed negative or near‐zero distortion index values, indicating reduced or unchanged deformation (Figure 3c, details in Table S4). Pairwise differences versus crunch were +0.26 ± 0.05, +0.19 ± 0.05, and +0.25 ± 0.05, respectively; all *p* < 0.001). However, given violations of model assumptions, additional non‐parametric paired Wilcoxon tests with Bonferroni correction were performed. These analyses showed consistent differences between crunch and the other exercises at both supraumbilical (all adjusted *p* ≤ 0.0097) and infraumbilical levels (all adjusted *p* ≤ 0.0011), supporting the robustness of the observed differences (Table S5).

Infraumbilically, crunch also showed positive distortion index values during the exercise (+0.30 ± 0.06), whereas ADIM, PFM, and de Gasquet showed minimal or negative changes (Figure 3D, details in Table S4). Non‐parametric Wilcoxon tests confirmed significant differences between crunch and the other exercises (all adjusted *p* ≤ 0.0011). These findings should be interpreted cautiously due to the limited variability and model fit limitations.

3. Thickness: Supraumbilically, the crunch was also the only exercise that showed negative thickness decrease values (−0.09 ± 0.05 mm), indicating increased LA thickness during exercise (Figure 3e, details in Table S4). It differed from ADIM (*p* = 0.05), PFM (*p* = 0.01) and de Gasquet (*p* = 0.01) (all other pairs ns [*p* ≥ 0.83]). No significant differences were found for the other exercises between the rest position and the thickness of the LA during exercise. Infraumbilically, thickness decrease values were small and close to zero across all exercises (crunch: −0.03 ;± 0.06 mm; ADIM: 0.00 ± 0.05 mm; PFM: −0.02 ± 0.05 mm; de Gasquet: 0.01 ± 0.06 mm), indicating no meaningful change in LA thickness, with no significant differences between exercises (Figure 3f) (all pairwise comparisons ns (*p* = 0.88–1.00).

In conclusion, even though the crunch reduced IRD the most supraumbilically, this change was not considered significant when compared with ADIM, PFM, and de Gasquet basic exercise. Additionally, the crunch produced greater distortion on the LA, both supra and infraumbilically. The results of the comparison between the different exercises are displayed in the Table 2.

**TABLE 2:** Comparisons of estimated marginal means as a function of the type of exercise according to Tukey's test.

Level	Outcome	Comparison	Estimate	95% CI	Adjusted *p*‐value	Effect size
Supraumbilical	IRD decrease	Crunch—ADIM	1.85	−0.44 to 4.14	0.34	1.67
		Crunch—PFM	2.61	0.55 to 4.67	0.07	2.50
		Crunch—de Gasquet	2.06	0.02 to 4.10	0.20	1.99
		ADIM—PFM	0.76	−1.22 to 2.74	0.87	0.76
		ADIM—de Gasquet	0.21	−1.67 to 2.09	0.10	0.22
		PFM—de Gasquet	−0.55	−2.41 to 1.31	0.94	−0.58
	LA thickness decrease	Crunch—ADIM	−0.11	−0.19 to −0.03	0.05	−2.60
		Crunch—PFM	−0.15	−0.23 to −0.07	0.01	−3.40
		Crunch—de Gasquet	−0.13	−0.21 to −0.05	0.01	−3.14
		ADIM—PFM	−0.04	−0.12 to 0.04	0.83	−0.84
		ADIM—de Gasquet	−0.02	−0.10 to 0.06	0.95	−0.56
		PFM—de Gasquet	0.01	−0.07 to 0.09	0.99	0.29
	Distortion index increase	Crunch—ADIM	0.26	0.16 to 0.36	< 0.001	5.36
		Crunch—PFM	0.19	0.09 to 0.29	< 0.001	3.96
		Crunch—de Gasquet	0.25	0.15 to 0.35	< 0.001	5.29
		ADIM—PFM	−0.07	−0.17 to 0.03	0.51	−1.39
		ADIM—de Gasquet	−0.004	−0.10 to 0.09	1.00	−0.08
		PFM—de Gasquet	0.06	−0.04 to 0.16	0.56	1.30
Infraumbilical	IRD decrease	Crunch—ADIM	0.48	−1.52 to 2.48	0.97	0.47
		Crunch—PFM	1.25	−0.75 to 3.25	0.61	1.23
		Crunch—de Gasquet	1.71	−0.29 to 3.71	0.34	1.68
		ADIM—PFM	0.77	−1.19 to 2.73	0.87	0.77
		ADIM—de Gasquet	1.23	−0.75 to 3.21	0.61	1.22
		PFM—de Gasquet	0.46	−1.50 to 2.42	0.97	0.46
	LA thickness decrease	Crunch—ADIM	−0.04	−0.16 to 0.08	0.93	−0.60
		Crunch—PFM	−0.01	−0.13 to 0.11	1.00	−0.20
		Crunch—de Gasquet	−0.05	−0.17 to 0.07	0.88	−0.74
		ADIM—PFM	0.02	−0.10 to 0.14	0.98	0.40
		ADIM—de Gasquet	−0.01	−0.13A to 0.11	1.00	−0.15
		PFM—de Gasquet	−0.03	−0.15 to 0.09	0.95	−0.55
	Distortion index increase	Crunch—ADIM	0.35	0.21 to 0.49	< 0.001	5.32
		Crunch—PFM	0.29	0.15 to 0.43	< 0.001	4.30
		Crunch—de Gasquet	0.30	0.16 to 0.44	< 0.001	4.51
		ADIM—PFM	−0.06	−0.18 to 0.06	0.78	−0.94
		ADIM—de Gasquet	−0.05	−0.17 to 0.07	0.87	−0.77
		PFM—de Gasquet	0.01	−0.11 to 0.13	1.00	0.17

*Note:* The *p*‐values, 95% confidence intervals (CI), and effect sizes (Cohen's d) are reported *p* [95% CI]; *d.*

Abbreviations: ADIM: Abdominal drawing‐in maneuver; IRD: inter‐recti distance; LA: linea alba; PFM: Pelvic floor muscle contraction.

### 3.2 Relationship Between IRD, LA Deformation, and TrA Activation

In the univariate analysis, an increase in TrA thickness during exercise was significantly associated with IRD, distortion index, and LA thickness, but only at the supraumbilical level. Multivariate analysis found that TrA thickness influenced IRD during exercise in both the crunch and the ADIM although the movement was performed in opposite directions. Notably, during the crunch, a lower increase in the TrA thickness was associated with a greater narrowing during the exercise. Figure 4 illustrates the interaction between IRD and TrA muscle thickening during the four exercises.

## 4 Discussion

Our research contributes to understanding the immediate effect of different exercises on abdominal wall morphology and function in parous women. Our findings indicate that (1) an increase in TrA muscle thickness during exercise was significantly associated with IRD, LA thickness, and the distortion index, but only at the supraumbilical level; (2) TrA activation affected IRD differently depending on the exercise: in the ADIM exercise, greater TrA thickening was associated with lower IRD, while in the crunch exercise lower TrA thickening was associated with greater IRD decrease, indicating greater narrowing; (3) no significant differences in IRD were observed between the exercises; (4) the crunch exercise produced a decrease in supraumbilical IRD but was accompanied by an increase in LA deformation, while the ADIM, PFM contraction, and basic de Gasquet exercise tended to reduce or maintain LA distortion while preserving its thickness.

### 4.1 Exercise‐Specific Differences (Crunch vs. TrA‐Based Maneuvers)

The crunch showed the largest supraumbilical IRD reduction during the exercise; however, this effect was not significant when compared to ADIM, PFM, or de Gasquet exercise, and it was accompanied by higher LA distortion at both supra and infraumbilical levels. These results are consistent with previous studies reporting a reduction in supraumbilical IRD during abdominal trunk‐flexion tasks (Gluppe et al. 2020; Mota et al. 2015; Pascoal et al. 2014; Sancho et al. 2015). In our sample, the crunch showed negative IRD decrease values infraumbilically, indicating widening although this was not statistically significant when compared to other exercises. This is consistent with the results of Sancho et al., who also did not observe significant changes in the infraumbilical region during crunch, but differs from those of Mota et al. and Gluppe et al. who observed a significant decrease(2020, 2015; Sancho et al. 2015). Overall, studies show a trend toward greater changes in the supraumbilical region than in the infraumbilical region.

Other studies in the literature have also investigated the behavior of the LA during an ADIM (Gluppe et al. 2020; Sancho et al. 2015) and a PFM contraction (Gluppe et al. 2020). While our findings are consistent with theirs at the infraumbilical level, this is not the case at the supraumbilical level. Gluppe et al. 2020; Sancho et al. 2015 reported an increase in IRD at the supraumbilical level during both exercises. Nevertheless, our study did not find significant differences at the supraumbilical level compared to the resting position. This difference could be explained by differences in the study population: our sample included multiparous women without a specific time since childbirth, while other studies included participants in the early postpartum period or nulliparous women. Differences in statistical analysis could also have influenced these results, including the incorporation of demographic and clinical covariates in our multivariate models.

While other exercises have evaluated the effect of hypopressive exercises on the behavior of the LA, no other study has specifically evaluated exercises based on de Gasquet's principle. Cuña‐Carrera evaluated the effect of hypopressive exercises and found that they did not appear to influence the IRD at any point (Da Cuña‐Carrera et al. 2021). However, in the study by Arranz Martín et al. (Arranz‐Martín et al. 2022), which also evaluated the effect of a hypopressive exercise, observed a reduction in IRD at the infraumbilical level. In their study, active hypopressive exercise tended to narrow the IRD below the umbilicus without inducing LA distortion (Arranz‐Martín et al. 2022). In this study, the women were 3 months postpartum, while in the Cuña‐Carrera study, the subjects were healthy. Findings from our study revealed no significant differences among the de Gasquet exercise, ADIM, and PFM contractions in relation to any exercise condition or measured outcome.

While previous research has examined changes in IRD and LA distortion during different tasks, the thickness of the LA has received little attention. LA thickness may provide additional information about the integrity of the connective tissue, since an exercise that excessively thins the LA could potentially affect its structural function. Baseline ultrasound studies described a mean resting LA thickness of approximately 3 mm near the umbilicus (Woxnerud et al. 2023). In our study, the crunch significantly increased LA thickness at the supraumbilical level, whereas no significant changes were observed during the ADIM, PFM, or de Gasquet exercises. This apparent thickening may reflect changes in connective tissue geometry during muscle activation rather than true structural hypertrophy. In contrast, the ADIM, PFM, and de Gasquet exercises appear to maintain tissue thickness while minimizing deformation.

### 4.2 Relationship Between IRD, LA Deformation, and TrA Activation

To our knowledge, no other study has assessed IRD and TrA thickening simultaneously during exercise. Remarkably, lower TrA thickening was associated with greater IRD decrease during crunch exercises, aligning with the TrA's role in minimizing LA deformation(Lee and Hodges 2016). Therefore, reduced activation of the TrA may lead to increased LA deformation, potentially causing the medial borders of the rectus abdominis to approximate and collapse—a phenomenon observed in the study by Lee et al. (2016). One example of this behavior is illustrated through in Video S1. Conversely, activation of the TrA muscle during the ADIM exercise was significantly associated with a reduction in IRD. It may seem contradictory that, while the TrA is anatomically oriented to pull the LA laterally and therefore theoretically increases the IRD, our results showed that its activation was associated with a decrease in IRD. In this context, it is important to consider the complex and non‐linear behavior of connective tissue, as well as the specific mechanics of each exercise. As described by van Wingerden et al., a gliding interface (with an interposed adipose layer) permits the posterior rectus sheath to move relative to the rectus abdominis when TrA is activated (van Wingerden et al. 2020). This phenomenon can reduce fascial slack and stiffen the posterior sheath/LA, effectively drawing the LA inward without visibly displacing the rectus laterally. To further confirm the behavior of the TrA during exercise, it would have been beneficial to measure not only the thickening of the muscle belly but also its lateral displacement using ultrasound.

### 4.3 Limitations and Future Directions

This observational study aims to understand the behavior of the LA during exercise by incorporating new outcomes in its assessment. However, it is necessary to acknowledge the limitations of some of the measurement tools that we employed. First, there is a potential variability due to the use of two different ultrasound systems. Although measurements showed high reliability, the absence of formal cross‐device calibration may introduce small systematic differences in absolute values, and findings should be interpreted accordingly. Also, although ultrasound is a valid method for evaluating IRD (Chiarello and McAuley 2013), this is not the case for the distortion index and LA thickness measurements. While the distortion index is considered an indicator of LA deformation (Beamish et al. 2019; Lee and Hodges 2016), more validation studies are necessary to establish its correlation with functional outcomes. Moreover, distortion index models showed deviations from normality and homoscedasticity assumptions, likely due to the limited variability of this outcome. Although non‐parametric sensitivity analyses yielded consistent results, distortion findings should be interpreted cautiously and confirmed in future studies.

While our findings should be interpreted with caution, this exploratory analysis lays the groundwork for future interventional studies. Future research should focus on the validation of robust and reliable outcomes for evaluating patients with DRA. Moreover, we believe that functional parameters should be combined with patient‐reported outcomes to capture patients' subjective experiences, thereby moving toward a more patient‐centered care model.

Future research should take this variability into account, considering the individual responses of each patient, in order to better understand their motor strategies and the impact of each exercise on abdominal wall function. Additionally, since different exercises may have different effects on intra‐abdominal pressure, it could be interesting to explore the combined effect of intra‐abdominal pressure during exercises and the comprehensive behavior of the LA.

## 5 Implications of Physiotherapy Practice

This study provides a detailed characterization of the immediate mechanical responses of the LA to different exercise conditions by simultaneously assessing IRD, LA deformation, thickness, and TrA behavior. These findings improve understanding of abdominal wall mechanical behavior during exercise but should not be interpreted as evidence of clinical effectiveness. DRA has been associated with outcomes including gastrointestinal symptoms, abdominal discomfort, altered body image, and reduced quality of life (Fuentes Aparicio et al. 2021). However, the extent to which specific morphological features of the LA are related to these outcomes remains unclear.

Previous studies have shown that IRD alone does not fully reflect LA mechanical behavior, as connective tissue deformation may vary independently of IRD (Lee and Hodges 2016). LA thickness may provide additional insight into its mechanical state, as connective tissues change thickness in response to loading, reflecting variations in tension and force transmission (Benjamin et al. 2008; Fung 1993). Because the LA is mechanically influenced by surrounding muscles, particularly the TrA through its aponeurotic insertions, its morphology also reflects muscle activation and connective tissue tension (Hodges et al. 2003; Maas and Sandercock 2010). Simultaneous assessment of thickness, deformation, and TrA activation may therefore improve characterization of abdominal wall mechanical responses, although their clinical significance remains uncertain.

Regional differences observed between supra‐ and infra‐umbilical levels further indicate that LA behavior is site‐specific, consistent with previous reports of anatomical and functional variability (Keshwani et al. 2018; Mota et al. 2015).This suggests that single‐site measurements may not represent the mechanical behavior of the entire structure.

Exercises integrating breathing, posture, and coordinated activation of the PFM and TrA, such as the de Gasquet basic exercise, were associated with limited deformation, maintenance of thickness, and minimal increase in IRD. These findings indicate that these exercises represent an additional approach capable of generating immediate mechanical responses of the LA comparable to those observed during TrA activation exercises and PFM exercises. However, these observations reflect only acute mechanical responses, and their relationship to clinical outcomes or comparative efficacy is unknown.

## Author Contributions

M.R.C. and L.F.A. contributed substantially to the conception and design of the work, acquisition and interpretation of the data, and were all involved in manuscript writing. B.d.G. contributed substantially to the interpretation of the data and critically revised the work for important intellectual content. All authors have approved the final version before publication and agree to be accountable for all aspects of the work in ensuring that questions related to the accuracy or integrity of any part of the work are appropriately investigated and resolved.

## Funding

The authors have nothing to report.

## Ethics Statement

The study complies with the Declaration of Helsinki and was approved by The Ethical Committee Comité de Protection des Personnes Ouest III (20.01.16.71857).

## Consent

All participants signed an informed consent form prior to their participation in the study. Any experiments on participants were undertaken with the understanding and appropriate informed consent. The photograph included in the manuscript features a researcher with blurred facial features to ensure anonymity. Written consent for publication of this image was obtained.

## Conflicts of Interest

M.R. and L.F. declare no conflicts of interest. B.G. discloses a potential conflict of interest as the developer of the method employed in one of the exercises tested in this study.

## Supporting information


**Figure S1:** Distribution of outcomes by postpartum duration. Boxplots of abdominal wall outcomes by postpartum duration. No meaningful differences were observed between groups, except for a small increase in infraumbilical distortion index in participants > 5 years postpartum. Overall, postpartum duration did not substantially influence morphological responses.

**Table S1:** Stratified univariate comparisons by postpartum duration.

**Table S2:** Sensitivity analysis including postpartum duration in mixed‐effects models.

**Table S3:** Univariate analyses of the effect of the independent variables on inter‐recti distance, linea alba thickness and distortion index.

**Table S4:** Comparison of inter‐recti distance decrease, linea alba thickness decrease, and distortion index increase during the four exercises.

**Table S5:** Non‐parametric pairwise comparisons of distortion index between exercises using paired Wilcoxon signed‐rank tests with Bonferroni correction.

**Video S1:** Behaviour of LA and rectus abdominis during crunch exercise. To view this video in the full‐text HTML version of the article, please visit https://onlinelibrary.wiley.com/doi/10.1002/pri.70185.

## Data Availability

Dataset is available at Rejano‐Campo, Montserrat; Fuentes‐Aparicio, Laura; de Gasquet, Bernadette (2025), “Linea alba response to exercise in parous women: research data from a cross‐sectional study”, Mendeley Data, V1, doi: 10.17632/2v288r7k2j.1.
