# Supplementary material for: Immediate Effect of Four Exercises on Linea Alba Thickness, Distortion and Inter‐Recti Distance in Parous Women
Source: Physiother Res Int. 2026 Mar 7;31(2):e70185. doi: 10.1002/pri.70185 (PMC12967262; doi:10.1002/pri.70185)

**Figure S1. Distribution of outcomes by postpartum duration. Boxplots of abdominal wall outcomes by postpartum duration**. No meaningful differences were observed between groups, except for a small increase in infraumbilical distortion index in participants >5 years postpartum. Overall, postpartum duration did not substantially influence morphological responses. Legend: EP_TENSION_SO_100: Supraumbilical thickness decrease; EP_TENSION_IO_100: Infraumbilical thickness decrease; DIR_RAC_SO_100: Supraumbilical IRD decrease; DIR_RAC_IO_100: Infraumbilical IRD decrease; DIST_AUM_SO_100: Supraumbilical distortion increase; DIST_AUM_IO_100: Infraumbilical distortion increase.


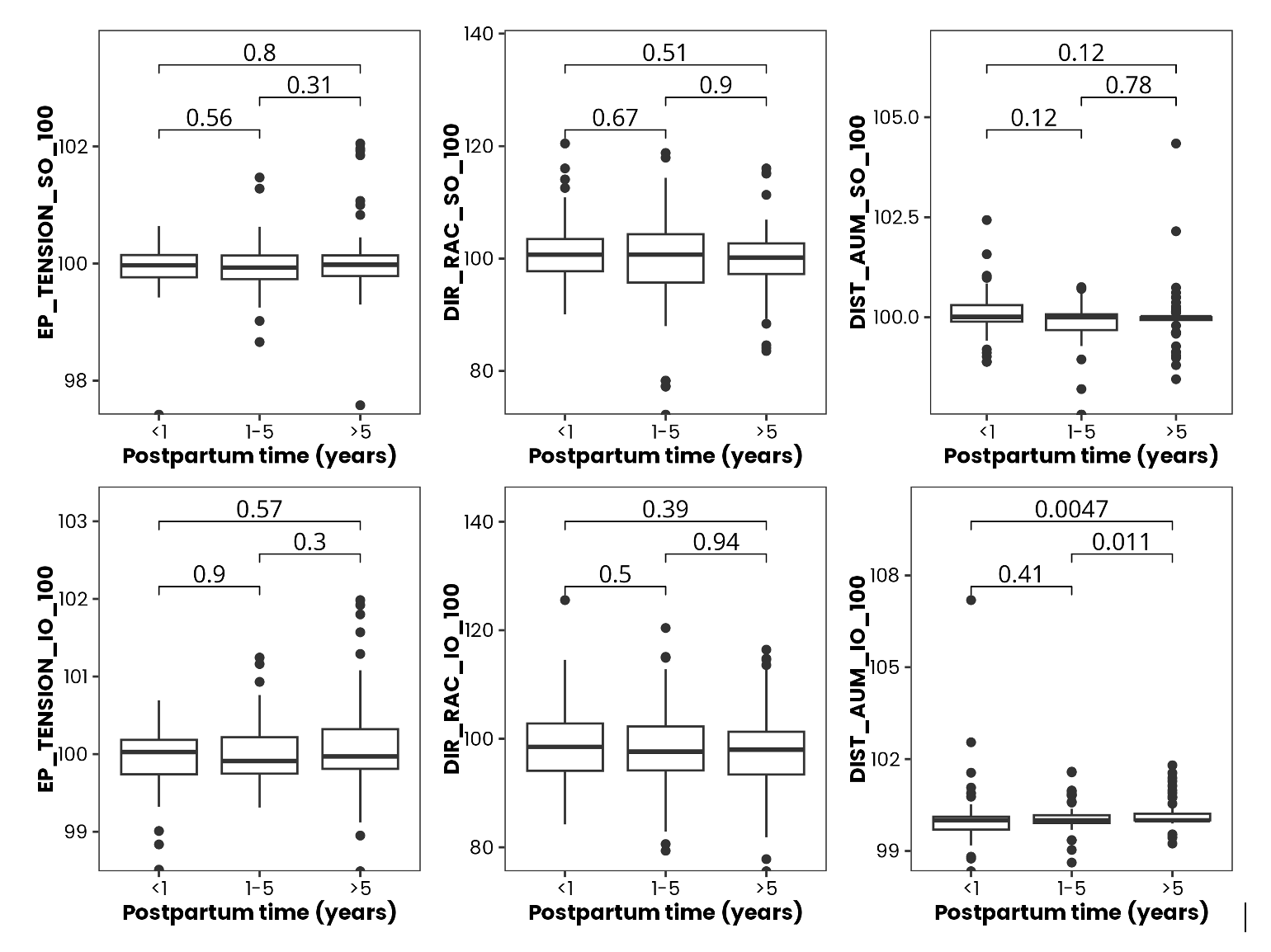

Supplement: Supplementary file 1 — Figure S1: Distribution of outcomes by postpartum duration. Boxplots of abdominal wall outcomes by postpartum duration. No meaningful differences were observed between groups, except for a small increase in infraumbilical distortion index in participants > 5 years postpartum. Overall, postpartum duration did not substantially influence morphological responses. [file PRI-31-e70185-s007.docx]
