# Supplementary material for: Immediate Effect of Four Exercises on Linea Alba Thickness, Distortion and Inter‐Recti Distance in Parous Women
Source: Physiother Res Int. 2026 Mar 7;31(2):e70185. doi: 10.1002/pri.70185 (PMC12967262; doi:10.1002/pri.70185)
Supplement: Supplementary file 2 — Table S1: Stratified univariate comparisons by postpartum duration. [file PRI-31-e70185-s006.docx]

# Table S1. Stratified univariate comparisons by postpartum duration.

## Outcome variables are expressed as percentage relative to rest (rest = 100). Values <100 indicate narrowing (IRD), thinning (linea alba thickness), or reduced deformation (distortion index), whereas values >100 indicate widening, thickening, or increased deformation. No consistent differences were observed between postpartum duration groups. Legend: IRD: Inter-recti distance

| Outcome | <1 | 1-5 | >5 | p.value |
| --- | --- | --- | --- | --- |
| Supraumbilical thickness decrease | 99.93 ± 0.45 (a) | 99.94 ± 0.43 (a) | 100.03 ± 0.56 (a) | 0,61 |
| Infraumbilical thickness decrease | 99.94 ± 0.42 (a) | 100.02 ± 0.42 (a) | 100.12 ± 0.59 (a) | 0,60 |
| Supraumbilical IRD decrease | 101.12 ± 6.35 (a) | 99.2 ± 9.42 (a) | 100.12 ± 5.35 (a) | 0,81 |
| Infraumbilical IRD decrease | 99.22 ± 6.96 (a) | 97.91 ± 8.4 (a) | 97.4 ± 7.92 (a) | 0,68 |
| Supraumbilical distortion increase | 100.1 ± 0.62 (a) | 99.84 ± 0.66 (a) | 99.99 ± 0.59 (a) | 0,22 |
| Infraumbilical distortion increase | 100.1 ± 1.2 (a) | 100.09 ± 0.5 (a) | 100.17 ± 0.41 (b) | < 0,01** |

## 
