# Supplementary material for: Immediate Effect of Four Exercises on Linea Alba Thickness, Distortion and Inter‐Recti Distance in Parous Women
Source: Physiother Res Int. 2026 Mar 7;31(2):e70185. doi: 10.1002/pri.70185 (PMC12967262; doi:10.1002/pri.70185)
Supplement: Supplementary file 3 — Table S2: Sensitivity analysis including postpartum duration in mixed‐effects models. [file PRI-31-e70185-s002.docx]

**Table S.2. Sensitivity analysis including postpartum duration in mixed-effects models.** Exercise coding: Crunch (reference), ADIM (abdominal drawing-in maneuver), PFM (pelvic floor muscle contraction), and De Gasquet basic exercise. Exercise-related coefficients remained stable after adjustment for postpartum duration, indicating that postpartum duration did not influence the main findings. Legend: IRD: Inter-recti distance; ADIM: abdominal drawing-in maneuver; PFM: pelvic floor muscle contraction; TrA: transversus abdominis muscle; IO: internal oblique.

|  |  |  |  |  |  |
| --- | --- | --- | --- | --- | --- |
|  |  | **Original model (included in the main text)** | | **Sensitivity analysis including postpartum time** | |
|  |  | **Estimate.x** | **p.value** | **Estimate.y** | **p.value** |
| **Supraumbilical thickness decrease** | |  |  |  |  |
|  | Intercept. | 0.02 | 0.76 | 0.06 | 0.57 |
|  | coactivation PFM-TrA | -0.22 | 0.01 | -0.23 | 0.01 |
|  | Supraumbilical distortion at rest | -0.10 | 0.01 | -0.10 | 0.01 |
|  | ADIM group | 0.11 | 0.01 | 0.11 | 0.01 |
|  | PFM group | 0.15 | 0.00 | 0.15 | 0.00 |
|  | de Gasquet group | 0.13 | 0.00 | 0.13 | 0.00 |
|  | years since delivery_1 | — | — | -0.00 | 0.99 |
|  | years since delivery | — | — | -0.06 | 0.52 |
| **Infraumbilical thickness decrease** | |  |  |  |  |
|  | Intercept. | -0.03 | 0.55 | -0.07 | 0.41 |
|  | Infraumbilical thickness at rest | 0.15 | 0.00 | 0.15 | 0.00 |
|  | Infraumbilical IRD decrease | -0.10 | 0.00 | -0.10 | 0.00 |
|  | ADIM group | 0.04 | 0.55 | 0.04 | 0.55 |
|  | PFM group | 0.01 | 0.84 | 0.01 | 0.84 |
|  | de Gasquet group | 0.05 | 0.46 | 0.05 | 0.45 |
|  | years since delivery_1 | — | — | 0.07 | 0.49 |
|  | years since delivery | — | — | 0.04 | 0.71 |
| **Supraumbilical DIR decrease** | |  |  |  |  |
|  | Intercept. | 3.32 | 0.03 | 2.41 | 0.23 |
|  | coactivation PFM-TrA | -1.69 | 0.34 | -1.63 | 0.37 |
|  | Supraumbilical IRD at rest | 3.08 | 0.00 | 3.30 | 0.00 |
|  | Supraumbilical distortion at rest | -0.44 | 0.54 | -0.49 | 0.51 |
|  | Supraumbilical TrA increase during exercise | -2.85 | 0.00 | -2.89 | 0.00 |
|  | ADIM group | -2.28 | 0.20 | -2.23 | 0.21 |
|  | PFM group | -3.44 | 0.05 | -3.43 | 0.05 |
|  | de Gasquet group | -2.20 | 0.20 | -2.17 | 0.21 |
|  | years since delivery_1 | — | — | 1.33 | 0.42 |
|  | years since delivery | — | — | 0.98 | 0.53 |
|  | coactivation PFM-TrA_ADIM | 0.85 | 0.67 | 0.86 | 0.67 |
|  | coactivation PFM-TrA_PFM | 1.65 | 0.40 | 1.66 | 0.40 |
|  | coactivation PFM-TrA_de Gasquet | 0.28 | 0.89 | 0.30 | 0.88 |
|  | Supraumbilical IRD at rest for ADIM group | -2.09 | 0.03 | -2.09 | 0.03 |
|  | Supraumbilical IRD at rest for PFM group | -1.95 | 0.03 | -1.94 | 0.03 |
|  | Supraumbilical IRD at rest for de Gasquet group | -2.32 | 0.01 | -2.34 | 0.01 |
|  | Supraumbilical distortion at rest for ADIM group | 2.93 | 0.00 | 2.95 | 0.00 |
|  | Supraumbilical distortion at rest for PFM group | 0.45 | 0.58 | 0.44 | 0.59 |
|  | Supraumbilical distortion at rest for de Gasquet group | 1.28 | 0.21 | 1.31 | 0.20 |
|  | Supraumbilical TrA increase during exercise for ADIM group | 4.10 | 0.00 | 4.12 | 0.00 |
|  | Supraumbilical TrA increase during exercise for PFM group | 2.66 | 0.01 | 2.69 | 0.01 |
|  | Supraumbilical TrA increase during exercise for de Gasquet group | 3.01 | 0.00 | 3.03 | 0.00 |
| **Infraumbilical DIR decrease** | |  |  |  |  |
|  | Intercept. | -0.98 | 0.32 | -1.13 | 0.52 |
|  | Infraumbilical IRD at rest | 5.43 | 0.00 | 5.51 | 0.00 |
|  | TrA thickness at rest | -0.40 | 0.69 | -0.44 | 0.67 |
|  | ADIM group | -0.48 | 0.64 | -0.48 | 0.64 |
|  | PFM group | -1.25 | 0.22 | -1.25 | 0.22 |
|  | de Gasquet group | -1.70 | 0.10 | -1.72 | 0.09 |
|  | years since delivery_1 | — | — | -0.23 | 0.92 |
|  | years since delivery | — | — | 0.56 | 0.79 |
|  | Infraumbilical IRD at rest for ADIM group | -3.29 | 0.00 | -3.30 | 0.00 |
|  | Infraumbilical IRD at rest for PFM group | -4.13 | 0.00 | -4.14 | 0.00 |
|  | Infraumbilical IRD at rest for de Gasquet group | -3.36 | 0.00 | -3.36 | 0.00 |
|  | TrA thickness at rest for ADIM group | 1.74 | 0.10 | 1.73 | 0.10 |
|  | TrA thickness at rest for PFM group | 2.07 | 0.05 | 2.06 | 0.05 |
|  | TrA thickness at rest for de Gasquet group | 1.17 | 0.26 | 1.17 | 0.27 |
| **Supraumbilical distortion increase** | |  |  |  |  |
|  | Intercept. | 0.16 | 0.00 | 0.27 | 0.00 |
|  | Supraumbilical distortion at rest | -0.22 | 0.00 | -0.24 | 0.00 |
|  | ADIM group | -0.26 | 0.00 | -0.26 | 0.00 |
|  | PFM group | -0.19 | 0.00 | -0.19 | 0.00 |
|  | de Gasquet group | -0.25 | 0.00 | -0.25 | 0.00 |
|  | years since delivery_1 | — | — | -0.09 | 0.22 |
|  | years since delivery | — | — | -0.20 | 0.01 |
| **Infraumbilical distortion increase** | |  |  |  |  |
|  | Intercept. | 0.30 | 0.00 | 0.25 | 0.01 |
|  | Infraumbilical distortion at rest | -0.23 | 0.00 | -0.23 | 0.00 |
|  | Infraumbilical thickness increase of IO during exercise | 0.12 | 0.00 | 0.12 | 0.00 |
|  | ADIM group | -0.35 | 0.00 | -0.34 | 0.00 |
|  | PFM group | -0.29 | 0.00 | -0.28 | 0.00 |
|  | de Gasquet group | -0.30 | 0.00 | -0.30 | 0.00 |
|  | years since delivery_1 | — | — | 0.09 | 0.39 |
|  | years since delivery | — | — | 0.05 | 0.65 |
