# Supplementary material for: Immediate Effect of Four Exercises on Linea Alba Thickness, Distortion and Inter‐Recti Distance in Parous Women
Source: Physiother Res Int. 2026 Mar 7;31(2):e70185. doi: 10.1002/pri.70185 (PMC12967262; doi:10.1002/pri.70185)
Supplement: Supplementary file 4 — Table S3: Univariate analyses of the effect of the independent variables on inter‐recti distance, linea alba thickness and distortion index. [file PRI-31-e70185-s005.docx]

**Table S3 .** **Univariate analyses of the effect of the independent variables on inter-recti distance, linea alba thickness and distortion index**. Significant results are indicated by asterisks. BMI: Body mass index, USP: Urinary Symptom Profile, VAS: visual analogue scale, PFM: pelvic floor muscles, TrA: transversus abdominis muscle, IO: internal oblique muscle, IRD: inter-recti distance, LA: linea alba, T: target variable.

| Outcomes | INTER-RECTI DISTANCE | | | | DISTORSION INDEX | | | | LINEA ALBA THICKNESS | | | | |
| --- | --- | --- | --- | --- | --- | --- | --- | --- | --- | --- | --- | --- | --- |
|  | **SUPRA** | | **INFRA** | | **SUPRA** | | **INFRA** | | **SUPRA** | | **INFRA** | | |
|  | **Chi** | ***p*** | **Chi** | ***p*** | **Chi** | ***p*** | **Chi** | ***p*** | **Chi** | ***p*** | **Chi** | ***p*** |  |
| Age | 0.31 | 0.58 | 0.03 | 0.87 | 2.4 | 0.12 | 0.16 | 0.69 | 0.95 | 0.33 | 0.03 | 0.86 |  |
| BMI | 0.1 | 0.76 | 0.06 | 0.81 | 0.86 | 0.35 | 0.6 | 0.44 | 0.64 | 0.42 | 1.51 | 0.22 |  |
| Type of sport | 3.16 | 0.20 | 1.43 | 0.49 | 0.24 | 0.89 | 3.13 | 0.21 | 2.33 | 0.31 | 0.89 | 0.64 |  |
| Number of deliveries | 0.67 | 0.41 | 1.45 | 0.23 | 2.58 | 0.11 | 0.01 | 0.91 | 1.54 | 0.21 | 1.61 | 0.2 |  |
| USP score | 1.16 | 0.28 | 0.14 | 0.71 | 0.13 | 0.72 | 0.03 | 0.87 | 0.01 | 0.91 | 0.03 | 0.87 |  |
| VAS pain score | 1.56 | 0.21 | 0.97 | 0.32 | 0.05 | 0.82 | 0.04 | 0.85 | 0.01 | 0.94 | 0.68 | 0.41 |  |
| PFM contraction present | 0.13 | 0.72 | 0.25 | 0.62 | 0.07 | 0.79 | 0.01 | 0.92 | 2.01 | 0.16 | 0.17 | 0.68 |  |
| Coactivation TrA-PFM present | 2.24 | 0.14 | 0.33 | 0.57 | 4.17 | **0.04*** | 1.1 | 0.3 | 4.29 | **0.04*** | 3.03 | 0.08 |  |
| TrA thickness at rest | 0.02 | 0.9 | 3.08 | 0.08 | 0.67 | 0.42 | 0.3 | 0.59 | 0.01 | 0.93 | 0.98 | 0.32 |  |
| IO thickness at rest | 0.52 | 0.47 | 0.02 | 0.89 | 0.04 | 0.84 | 1.37 | 0.24 | 1.68 | 0.2 | 1.42 | 0.23 |  |
| IRD at rest | 11.82 | **< 0.001*** | 14.22 | **< 0.001*** | 2.51 | 0.11 | 5.27 | **0.02*** | 5.12 | **0.02*** | 7.43 | **0.01*** |  |
| LA thickness at rest | 1.8 | 0.18 | 1.22 | 0.27 | 0.2 | 0.66 | 1.33 | 0.25 | 1.8 | 0.18 | 16.89 | **< 0.001*** |  |
| LA distortion at rest | 4.07 | **0.04*** | 0.35 | 0.56 | 57.29 | **< 0.001*** | 38.54 | **< 0.001*** | 4.55 | **0.03*** | 0.57 | 0.45 |  |
| TrA thickness increase during exercise | 4.83 | **0.03*** | 1.48 | 0.22 | 11.06 | **< 0.001*** | 0.45 | 0.5 | 5.47 | **0.02*** | 0 | 0.99 |  |
| IO thickness increase during exercise | 0.34 | 0.56 | 1.17 | 0.28 | 2.70 | 0.1 | 23.28 | **< 0.001*** | 1.58 | 0.21 | 0.65 | 0.42 |  |
| IRD decrease during exercise | T | T | T | T | 0.01 | 0.91 | 3.18 | 0.08 | 7.71 | **0.01*** | 14.76 | **< 0.001*** |  |
| Type of exercise | 19.20 | **< 0.001*** | 2.64 | 0.45 | 36.4 | **< 0.001*** | 43.18 | **< 0.001*** | 15.05 | **0.002*** | 1.42 | 0.7 |  |
