# Supplementary material for: Immediate Effect of Four Exercises on Linea Alba Thickness, Distortion and Inter‐Recti Distance in Parous Women
Source: Physiother Res Int. 2026 Mar 7;31(2):e70185. doi: 10.1002/pri.70185 (PMC12967262; doi:10.1002/pri.70185)
Supplement: Supplementary file 5 — Table S4: Comparison of inter‐recti distance decrease, linea alba thickness decrease, and distortion index increase during the four exercises. [file PRI-31-e70185-s001.docx]

**Table S4. Comparison of inter-recti distance decrease, linea alba thickness decrease, and distortion index increase during the four exercises.** Estimated marginal means with their standard errors, and 95% confidence intervals (CI) are presented, EMM ± SE [CI 95%].

Different letters (a, b) denote statistically significant differences between the exercises. If two exercises have the same letter, it indicates there is no significant difference in that metric between those exercises.

| Level / Variable | Crunch | ADIM | PFM | De Gasquet basic exercise |
| --- | --- | --- | --- | --- |
| Supraumbilical level |  |  |  |  |
| IRD decrease (mm) | 2.48 ± 0.96 [0.59 – 4.37]ᵃ | 0.63 ± 0.89 [–1.13 – 2.39]ᵃ | –0.13 ± 0.87 [–1.85 – 1.58]ᵃ | 0.42 ± 0.83 [–1.23 – 2.06]ᵃ |
| LA thickness decrease (mm) | –0.09 ± 0.05 [–0.18 – 0.01]ᵃ | 0.02 ± 0.05 [–0.07 – 0.12]ᵇ | 0.06 ± 0.05 [–0.04 – 0.15]ᵇ | 0.05 ± 0.05 [–0.05 – 0.14]ᵇ |
| Distortion index increase | 0.16 ± 0.04 [0.07 – 0.24]ᵇ | –0.10 ± 0.04 [–0.18 – –0.02]ᵃ | –0.04 ± 0.04 [–0.12 – 0.05]ᵃ | –0.10 ± 0.04 [–0.18 – –0.02]ᵃ |
| Infraumbilical level |  |  |  |  |
| IRD decrease (mm) | –0.98 ± 0.98 [–2.92 – 0.97]ᵃ | –1.45 ± 0.97 [–3.37 – 0.46]ᵃ | –2.22 ± 0.97 [–4.14 – –0.31]ᵃ | –2.68 ± 0.97 [–4.60 – –0.77]ᵃ |
| LA thickness decrease (mm) | –0.03 ± 0.06 [–0.14 – 0.08]ᵃ | 0.00 ± 0.05 [–0.10 – 0.11]ᵃ | –0.02 ± 0.05 [–0.13 – 0.09]ᵃ | 0.01 ± 0.06 [–0.09 – 0.12]ᵃ |
| Distortion index increase | 0.30 ± 0.06 [0.09 – 0.41]ᵃ | –0.05 ± 0.05 [–0.15 – 0.06]ᵃ | 0.01 ± 0.05 [–0.09 – 0.12]ᵃ | 0.00 ± 0.05 [–0.10 – 0.11]ᵃ |
