# Supplementary material for: Immediate Effect of Four Exercises on Linea Alba Thickness, Distortion and Inter‐Recti Distance in Parous Women
Source: Physiother Res Int. 2026 Mar 7;31(2):e70185. doi: 10.1002/pri.70185 (PMC12967262; doi:10.1002/pri.70185)
Supplement: Supplementary file 6 — Table S5: Non‐parametric pairwise comparisons of distortion index between exercises using paired Wilcoxon signed‐rank tests with Bonferroni correction. [file PRI-31-e70185-s003.docx]

**Table S5.** Non-parametric pairwise comparisons of distortion index between exercises using paired Wilcoxon signed-rank tests with Bonferroni correction. ADIM: Abdominal Drawing-in maneuver, PFM: Pelvic floor muscle

| Comparison | Supraumbilical adjusted p | Infraumbilical adjusted p |
| --- | --- | --- |
| Crunch vs ADIM | 0.0002 | 0.0005 |
| Crunch vs PFM | 0.0097 | 0.0001 |
| Crunch vs de Gasquet | 0.0014 | 0.0011 |
| ADIM vs PFM | 0.2526 | 1.000 |
| ADIM vs de Gasquet | 1.000 | 1.000 |
| PFM vs de Gasquet | 1.000 | 1.000 |
